# Organization of Psychosocial Processes in Metropolitan and Non-Metropolitan Regions

**DOI:** 10.65773/ure.1.1.68

**Published:** 2025-10-30

**Authors:** Shervin Assari, John Ashley Pallera

**Affiliations:** 1 Department of Psychiatry, Charles R. Drew University of Medicine and Science, Los Angeles, CA, USA; 2 Marginalization-related Diminished Returns (MDRs) Center, Los Angeles, CA, USA; 3 College of Medicine, Charles R. Drew University of Medicine and Science, Los Angeles, CA, USA

**Keywords:** adolescence, psychosocial factors, emotional well-being, social connectedness, behavioral risk, network analysis, metropolitan areas, non-metropolitan areas, geographic context

## Abstract

**Background:**

Adolescents’ social, emotional, and behavioral characteristics do not operate in isolation but form interconnected systems that shape well-being. These processes may differ across geographic settings, particularly between metropolitan (MSA) and non-metropolitan (non-MSA) environments, which vary in social norms, community resources, and daily experiences. However, little research has examined how these psychosocial factors are organized as part of a broader system in different environments.

**Objectives:**

This study compared the structure of psychosocial networks among adolescents living in MSA and non-MSA settings. We aimed to identify whether social, emotional, and behavioral constructs cluster differently across environments and whether specific constructs show stronger influence or bridging roles within each network.

**Methods:**

Data were drawn from the 2023 Monitoring the Future survey of U.S. adolescents. Twelve psychosocial and behavioral constructs were examined, including emotional distress, self-esteem, positive well-being, social connectedness, sensation seeking, boredom, problem behaviors, and perceived parental and peer disapproval of substance use, and substance use. Separate correlation-based networks were generated for MSA and non-MSA youth. System-level measures (average degree, betweenness, eigenvector centrality) were used to assess overall structure, and node-level metrics identified constructs serving as central or bridging elements within each network.

**Results:**

Emotional distress, sensation seeking, and problem behaviors played stronger bridging roles in non-MSA than MSA environments. In contrast, MSA networks were more evenly distributed, with positive well-being, social connectedness, and parental or peer disapproval showing stronger central influence.

**Conclusions:**

Different organization of psychosocial processes across environments can inform context-sensitive strategies to support adolescent well-being.

## 1. Introduction

Adolescence is a period marked by major social, emotional, and behavioral changes [1–6]. During these years, young people develop patterns of coping, interacting, and engaging with their environments, and these patterns may have long-term consequences for well-being [7]. Emotional states such as anxiety, hopelessness, and positive well-being, as well as social factors like connectedness and perceived support, do not operate independently [8,9]. Instead, these elements tend to coexist and influence one another, reflecting a broader system of psychosocial functioning [10,11].

Because these psychosocial processes are interconnected, changes in one area may be associated with shifts in others [12]. For example, boredom or sensation seeking may relate to problem behaviors, while strong social connectedness may accompany better emotional functioning [13]. Such relationships mean that adolescents’ social, emotional, and behavioral profiles may be better understood not as isolated traits but as components of a larger, interrelated system [14].

Importantly, the way these processes coexist may differ across environmental contexts [15]. Metropolitan (MSA) and non-metropolitan (non-MSA) settings vary in community structures, social norms, access to resources, and exposure to stressors [16–18]. Urban and suburban areas often offer more opportunities for social engagement, extracurricular activities, and access to mental health services [19–28]. In contrast, youth in non-MSA environments may experience fewer structured activities, different social expectations, or limited access to supportive services. These contextual differences may shape how emotional, social, and behavioral constructs relate to one another.

Although prior research has compared mental health or behavioral outcomes across rural and urban settings [26,27,29–33], much of this work focuses on individual variables rather than the ways such variables cluster and interact. As a result, less is known about how the structure of psychosocial relationships differs across geographic contexts [34]. Understanding these structural differences may help clarify why certain challenges appear more common in some environments and how different settings might shape the organization of adolescents’ emotional and behavioral development [35].

Network or system-oriented approaches offer a way to examine these broader relational patterns [36–39]. Instead of treating each psychosocial construct separately, network methods map how constructs are linked, identify which ones appear most central, and highlight which factors serve as bridges across different parts of the system. Applying these methods to MSA and non-MSA youth may provide insight into whether the interconnectedness of psychosocial processes differs by environment.

### 1.1. Objectives

The present study aims to compare adolescents living in MSA and non-MSA settings on the structure of their social, emotional, and behavioral networks. By examining differences in system-level cohesion and node-level roles, the analysis may offer a fuller understanding of how environmental context shapes the organization of psychosocial functioning during adolescence.

## 2. Methods

### 2.1. Study Design and Data Source

This study used data from the 2023 Monitoring the Future (MTF) survey [40–48], a national, school-based study of U.S. adolescents. MTF follows a repeated cross-sectional design and collects information during regular school hours using anonymous questionnaires. The public-use data are fully deidentified and allow secondary analysis without direct contact with participants. For this analysis, we used the file that includes 8th- and 10th-grade students, as these years represent a period when many social, emotional, and behavioral patterns begin to take more stable form.

### 2.2. Sampling and Participants

MTF relies on multistage probability sampling. Geographic areas are selected first, followed by schools within those areas, and then classrooms inside sampled schools. Students receive either paper or tablet-based questionnaires, depending on school preference.

The analytic sample included all adolescents with valid data on metropolitan statistical area (MSA) status and the psychosocial, social, and behavioral constructs studied here. Students missing any of these variables were excluded from analysis. The final sample included youth from both MSA and non-MSA environments and reflected a wide range of sociodemographic backgrounds.

### 2.3. Measures

#### MSA Status.

Residential environment was measured by an item indicating whether the student lived in a metropolitan statistical area. Responses were coded as MSA or non-MSA. The non-MSA category included rural, small-town, farm, and other non-metropolitan areas.

Psychosocial, Emotional, and Behavioral Constructs. All constructs came from standard MTF items. Higher scores reflect higher levels of each construct unless noted otherwise. Hopelessness was drawn from an item assessing how often the student felt that future outcomes seemed discouraging or without purpose. Anxiety/worry measured the frequency of feeling nervous, tense, or unable to relax. Self-esteem reflected feelings of personal worth, confidence, and self-acceptance. Positive well-being captured general emotional balance, optimism, and satisfaction. Social connectedness represented feelings of belonging, closeness to others, and support from peers. Sensation seeking reflected tendencies toward excitement-seeking, risk-taking, and a desire for novel experiences. Boredom measured how often the student felt uninterested, restless, or disengaged from daily life. Problem behaviors captured involvement in behaviors such as rule-breaking or other actions that reflect behavioral dysregulation. Friends’ disapproval of drugs assessed how strongly close friends would disapprove of substance use. Higher scores reflect greater social discouragement. Parents’ disapproval of drugs measured parental norms and expectations against substance use. Higher values reflect stronger disapproval. Substance use was measured using lifetime (“ever use”) indicators for the following substances: nicotine, alcohol, marijuana, LSD, psychedelics, amphetamines, barbiturates, tranquilizers, heroin, inhalants, and narcotics.

#### Sociodemographic and Family Characteristics.

Grade (8th or 10th), sex, school region, and parental presence were included for descriptive comparisons. Parental presence was coded for mother and father separately. Parental education was reported on a six-level scale ranging from less than high school to graduate or professional degrees. Higher values indicated higher educational attainment.

### 2.4. IRB and Ethical Considerations

This project used publicly available, fully deidentified MTF data and therefore qualified as non-human subjects research under federal regulations. The University of Michigan Survey Research Center, which conducts MTF, obtained full IRB approval for all data collection procedures. In the original study, students provided assent, and parents or guardians provided consent according to local school policies. No new data were collected for the present analysis.

### 2.5. Analytic Strategy

We began by comparing MSA and non-MSA adolescents on demographic and family characteristics using chi-square tests for categorical variables and t-tests for continuous variables. To examine how constructs related to one another, we calculated Spearman correlations separately for MSA and non-MSA students. Absolute values of these correlations were used to construct networks, as they reflect the strength of relationships regardless of direction. Networks were created for each group using constructs as nodes and absolute correlations as edges. System-level metrics were then computed, including average degree (the number of connections per construct), betweenness centrality (how often a construct serves as a bridge linking different parts of the network), and eigenvector centrality (how connected a construct is to other influential constructs). Node-level metrics were calculated for each construct to show which emotional, social, or behavioral factors held more central, bridging, or influential positions within each environment. All descriptive analyses were conducted in Stata, and network models were generated using Python’s standard scientific computing libraries.

## 3. Results

Table 1 summarizes sample characteristics for the overall population and separately for adolescents living in MSA and non-MSA areas. Grade distribution differed across settings. Overall, 42.3% of the sample were 8th graders and 57.7% were 10th graders. Among MSA youth, 39.2% were in 8th grade and 60.8% were in 10th grade, whereas in non-MSA areas 51.0% were in 8th grade and 49.0% were in 10th grade (p < 0.001).

Sex distribution also varied between the two settings. In the full sample, 32.2% were male and 39.9% female, with 27.9% missing or unspecified. Among MSA adolescents, 34.6% were male and 41.1% were female, while in non-MSA areas 25.4% were male and 36.4% were female (p < 0.001).

Patterns of parental presence showed high levels of father and mother presence in both groups. Father presence was reported by 77.3% overall, 77.0% in MSA, and 78.0% in non-MSA areas (p = 0.288). Mother presence was reported by 89.6% overall, 90.5% in MSA, and 87.2% in non-MSA settings (p < 0.001).

Parental education levels also showed numeric differences across groups. Average father education was 4.01 (SD = 1.45) in the full sample, 4.10 (SD = 1.47) among MSA youth, and 3.75 (SD = 1.35) in non-MSA youth (p < 0.001). Mean mother education followed a similar pattern at 4.42 (SD = 1.35) overall, 4.49 (SD = 1.35) in MSA, and 4.22 (SD = 1.33) in non-MSA settings (p < 0.001).

Table 2 presents descriptive characteristics of the total study sample and by MSA status. Across the full sample, mean values for psychosocial and behavioral variables ranged from 1.17 for problem behaviors to 3.82 for health behaviors, with standard deviations between 0.46 and 1.25. When comparing MSA and non-MSA groups, mean values for each construct showed small numeric differences. For example, health behaviors averaged 3.86 in MSA and 3.74 in non-MSA youth, while hopelessness averaged 3.28 in MSA and 3.24 in non-MSA. Similar patterns were observed across other variables, with means for sensation seeking, boredom, and social connectedness differing slightly between settings (Table 2).

Table 3 summarizes the bivariate Spearman correlations among the twelve psychosocial and behavioral variables for the full sample. Correlations ranged in magnitude from near zero to moderate levels, with the strongest associations observed between hopelessness and anxiety/worry (ρ = 0.63) and between problem behaviors and sensation seeking (ρ = 0.48). Social connectedness showed negative correlations with several indicators of emotional distress, including hopelessness (ρ = −0.34) and anxiety/worry (ρ = −0.28). Positive well-being was positively related to self-esteem (ρ = 0.50) and negatively related to hopelessness (ρ = −0.42) and anxiety/worry (ρ = −0.36).

Table 3 provides separate correlation matrices for MSA and non-MSA adolescents. The overall pattern of correlations was similar across the two groups, though individual coefficients varied slightly. In both settings, the largest correlations occurred between hopelessness and anxiety (MSA ρ = 0.63; non-MSA ρ = 0.63) and between problem behaviors and sensation seeking (MSA ρ = 0.45; non-MSA ρ = 0.51). Social connectedness showed inverse correlations with emotional distress in both groups. Positive well-being and self-esteem demonstrated consistently positive correlations across MSA and non-MSA settings. All coefficients appear in Table 3.

System-level network metrics for MSA and non-MSA adolescents are shown in Table 4. The mean absolute correlation across all psychosocial connections was 0.202 in the MSA group and 0.213 in the non-MSA group. The average degree, defined as the number of connections per construct at the |ρ| > 0.10 threshold, was 7.33 for MSA youth and 7.50 for non-MSA youth. Mean betweenness centrality values were 0.033 in the MSA group and 0.032 in the non-MSA group. Mean eigenvector centrality values were similar across the two groups, measured at 0.280 in MSA settings and 0.279 in non-MSA settings.

As shown in Table 5, node-level centrality values were comparable across MSA and non-MSA networks. Degree centrality ranged from 3 to 11 in both settings. Positive well-being showed the highest degree in both groups (11 in MSA and 11 in non-MSA), while social connectedness showed the lowest degree (3 in both groups). Several variables had the same degree across settings, including hopelessness (9 vs. 9), self-esteem (9 vs. 9), and anxiety/worry (8 vs. 8).

Betweenness centrality values were low overall, ranging from 0.000 to 0.140 in MSA and from 0.000 to 0.123 in non-MSA areas. Positive well-being had the highest betweenness in the MSA group (0.140), while the substance -use index showed the highest betweenness in the non-MSA group (0.123). Some variables showed minimal betweenness in both settings, including social connectedness (0.000 in both) and sensation seeking (0.003 in MSA; 0.011 in non-MSA).

Eigenvector centrality values showed a similar range across environments, from 0.119 to 0.379 in MSA and from 0.118 to 0.368 in non-MSA. Positive well-being had the highest eigenvector value in the MSA group (0.379), while the substance-use index had the highest value in the non-MSA group (0.368). The lowest eigenvector values were observed for social connectedness (0.119 MSA; 0.118 non-MSA).

## 4. Discussion

The present study examined whether adolescents living in metropolitan (MSA) and non-metropolitan (non-MSA) environments differ in how their social, emotional, and behavioral characteristics are organized as interconnected systems. Rather than focusing on individual variables, we mapped how constructs such as anxiety, hopelessness, self-esteem, positive well-being, social connectedness, boredom, sensation seeking, and problem behaviors relate to one another within each context. The findings suggest that although adolescents in both settings show similar levels of overall connectivity, the structure and organization of their psychosocial networks differ in meaningful ways.

Non-MSA adolescents showed higher overall betweenness centrality than MSA youth, suggesting a network more reliant on a small number of constructs to link different parts of the psychosocial system. Adolescents’ psychosocial systems may differ by geographic context [49,50]. We found that non-MSA youth appear to have networks shaped more strongly by emotional and behavioral vulnerabilities, whereas MSA youth show systems influenced more by social and emotional protective factors.

System-level comparisons showed that non-MSA adolescents had higher betweenness centrality, indicating that their psychosocial systems rely more heavily on a small set of constructs to link different parts of the network. This implies a more segmented structure, in which emotional distress and behavioral dysregulation may carry more responsibility for holding the system together. Such a pattern may reflect contextual differences in access to social, community, or emotional resources. In environments with fewer supports, certain constructs may take on larger bridging roles because there are fewer alternative pathways connecting social and emotional domains.

Node-level results supported these observations. In non-MSA settings, constructs such as problem behaviors, sensation seeking, anxiety, and hopelessness appeared as central connectors that linked emotional and behavioral domains. These findings suggest that when these risk-related constructs are present, they may influence a wider range of psychosocial characteristics. Although the analysis cannot determine directionality or causality, this pattern may reflect a system that is more vulnerable to cascading effects. A shift in emotional or behavioral functioning in non-MSA contexts may be associated with broader changes across the psychosocial network.

In contrast, the psychosocial structure among MSA adolescents was more evenly distributed. Constructs related to positive well-being, social connectedness, and perceived disapproval or support from parents and friends had stronger influence in MSA networks. This is consistent with environments that offer more opportunities for engagement, greater diversity in peer interactions, and potentially more access to counseling or school-based supports. The structure observed among MSA youth suggests that the system is shaped more by the presence of protective factors rather than a reliance on key risk-related constructs.

These findings may have practical implications for understanding how context shapes adolescents’ psychosocial functioning. In non-MSA settings, stronger influence from emotional distress and behavioral dysregulation suggests that early signs of boredom, sensation seeking, anxiety, or problem behaviors may be more consequential, as they appear more central to the broader psychosocial system. Interventions that strengthen coping skills, emotional regulation, and structured engagement may be particularly useful in these environments.

Tailoring prevention and intervention strategies to the structural organization of psychosocial processes, rather than only their levels, may be a promising approach to supporting adolescents across different environments.

For MSA youth, the stronger centrality of positive well-being, social connectedness, and perceived support suggests that reinforcing these existing strengths may help maintain overall psychosocial balance. Programs that build community belonging, peer support, and parental engagement may therefore have broader system-level effects in metropolitan areas.

Our findings align with studies suggesting that rural or non-metropolitan settings often differ from metropolitan ones in the availability of social opportunities, mental health resources, and contextual supports[26,27,31,33,51,52]. Prior work has shown that youth in smaller communities may face different stressors and may experience greater isolation, which could contribute to stronger clustering around emotional and behavioral risk factors [24,,27,31,51,53]. At the same time, studies of urban youth often highlight the role of peer connectedness and emotional well-being in shaping adjustment, which aligns with the centrality of protective constructs observed among MSA adolescents. The current findings extend this literature by examining how these factors relate to one another at the structural, system-wide level.

### 4.1. Limitations and Strengths

Several limitations should be noted. The analysis is cross-sectional, so it cannot determine the directionality or temporal sequencing of relationships among constructs. All measures were self-reported, which may introduce reporting bias. The measure used to classify MSA versus non-MSA settings is broad and cannot capture the full range of rural, small-town, suburban, or urban distinctions. Network models rely on observed correlations, which reflect associations rather than deeper causal processes. Finally, the public-use dataset limits access to some contextual or family-level variables that may further differentiate psychosocial networks across environments.

This study benefits from a large, nationally representative dataset and the inclusion of a wide array of psychosocial constructs. Using a network approach allowed us to examine how emotional, social, and behavioral factors are connected rather than treating each construct independently. Comparing MSA and non-MSA youth within the same analytic framework provides insight into how environmental context may shape the organization of psychosocial functioning.

### 4.2. Future Research

More longitudinal work is needed to understand how psychosocial networks evolve during adolescence and whether the structural differences observed here persist over time. Future studies may benefit from more detailed geographic variables, including rurality ratings, economic indicators, or community characteristics. Exploring differences by sex, socioeconomic status, race and ethnicity, and other social contexts could provide a more nuanced understanding of how networks operate across diverse groups. Multilayer network models that incorporate peers, family dynamics, school climate, and environmental stressors would also deepen our understanding of adolescent development.

## 5. Conclusion

This study suggests that adolescents’ social, emotional, and behavioral characteristics are organized differently across metropolitan and non-metropolitan environments. Non-MSA youth showed systems more heavily anchored by emotional distress, behavioral dysregulation, and sensation seeking, whereas MSA youth exhibited networks influenced more strongly by social connectedness and emotional well-being. Although overall connectivity was similar, the structural roles played by specific constructs differed across contexts. Recognizing these contextual differences may help guide prevention and support strategies that align with the unique psychosocial organization of adolescents’ lives.

## Figures and Tables

**Table 1.** Sample Characteristics Overall and by MSA Status

Category	Overall	MSA	Non-MSA	p-value

Grade				
8th grade	6004 (42.3%)	4106 (39.2%)	1898 (51.0%)	<0.001
10th grade	8198 (57.7%)	6376 (60.8%)	1822 (49.0%)	
Sex				
Male	4575 (32.2%)	3629 (34.6%)	946 (25.4%)	<0.001
Female	5665 (39.9%)	4311 (41.1%)	1354 (36.4%)	
	3962 (27.9%)	2542 (24.3%)	1420 (38.2%)	
Father presence				
Father present	10972 (77.3%)	8071 (77.0%)	2901 (78.0%)	0.288
Father not present	2459 (17.3%)	1846 (17.6%)	613 (16.5%)	
Mother presence				
Mother present	12729 (89.6%)	9486 (90.5%)	3243 (87.2%)	<0.001
Mother not present	702 (4.9%)	431 (4.1%)	271 (7.3%)	
	Mean (SD)	Mean (SD)	Mean (SD)	
Father education	4.01 (1.45)	4.10 (1.47)	3.75 (1.35)	<0.001
Mother education	4.42 (1.35)	4.49 (1.35)	4.22 (1.33)	<0.001

**Table 2.** Psychosocial, Emotional, and Behavioral Constructs Overall and by MSA

Variable	Overall	MSA	Non-MSA	p-value

Health behaviors	3.82 (0.97)	3.83 (0.97)	3.77 (0.99)	0.070
Substance-use	0.07 (0.11)	0.06 (0.10)	0.07 (0.12)	<0.001
Problem behaviors	1.17 (0.46)	1.18 (0.48)	1.15 (0.41)	0.001
Positive well-being	2.43 (0.96)	2.43 (0.95)	2.41 (0.97)	0.158
Hopelessness	2.35 (1.06)	2.35 (1.06)	2.34 (1.07)	0.627
Self-esteem	3.35 (0.72)	3.36 (0.72)	3.31 (0.73)	0.014
Anxiety	3.27 (1.25)	3.28 (1.24)	3.23 (1.29)	0.137
Social connectedness	3.45 (0.81)	3.46 (0.82)	3.41 (0.78)	0.161
Sensation seeking	2.89 (1.13)	2.89 (1.11)	2.91 (1.19)	0.544
Boredom	3.37 (1.23)	3.38 (1.20)	3.36 (1.31)	0.726
Friends’ disapproval of drugs	2.46 (0.64)	2.48 (0.63)	2.42 (0.67)	0.009
Parents’ disapproval of drugs	2.55 (0.67)	2.56 (0.66)	2.51 (0.69)	0.031

**Table 3.** Spearman Correlations Among Psychosocial Constructs

Variable	Health behaviors	Substance-use	Problem behaviors	Positive well-being	Hopelessness	Self-esteem	Anxiety	Social connected ness	Sensation seeking	Boredom	Friends’ disapproval of drugs	Parents’ disapproval of drugs

All Health behaviors	1.00	−0.11*	−0.05	0.35*	−0.23*	0.18*	−0.13*	0.23*	−0.04*	−0.02	0.09*	0.11*
Substance-use	−0.11*	1.00	0.29*	−0.14*	0.17*	−0.17*	0.17*	−0.11*	0.29*	0.21*	−0.12*	−0.13*
Problem behaviors	−0.05	0.29*	1.00	−0.11*	0.18*	−0.17*	0.18*	−0.05*	0.36*	0.30*	−0.08*	−0.10*
Positive well-being	0.35*	−0.14*	−0.11*	1.00	−0.46*	0.43*	−0.28*	0.40*	−0.12*	−0.17*	0.16*	0.18*
Hopelessness	−0.23*	0.17*	0.18*	−0.46*	1.00	−0.46*	0.41*	−0.27*	0.19*	0.25*	−0.11*	−0.13*
Self-esteem	0.18*	−0.17*	−0.17*	0.43*	−0.46*	1.00	−0.35*	0.33*	−0.19*	−0.25*	0.12*	0.13*
Anxiety	−0.13*	0.17*	0.18*	−0.28*	0.41*	−0.35*	1.00	−0.17*	0.16*	0.24*	−0.08*	−0.08*
Social connectedness	0.23*	−0.11*	−0.05*	0.40*	−0.27*	0.33*	−0.17*	1.00	−0.07*	−0.14*	0.13*	0.14*
Sensation seeking	−0.04*	0.29*	0.36*	−0.12*	0.19*	−0.19*	0.16*	−0.07*	1.00	0.33*	−0.09*	−0.09*
Boredom	−0.02	0.21*	0.30*	−0.17*	0.25*	−0.25*	0.24*	−0.14*	0.33*	1.00	−0.03	−0.04*
Friends’ disapproval of drugs	0.09*	−0.12*	−0.08*	0.16*	−0.11*	0.12*	−0.08*	0.13*	−0.09*	−0.03	1.00	0.53*
Parents’ disapproval of drugs	0.11*	−0.13*	−0.10*	0.18*	−0.13*	0.13*	−0.08*	0.14*	−0.09*	−0.04*	0.53*	1.00
MSA												
Health behaviors	1.00	−0.09*	−0.04*	0.35*	−0.21*	0.17*	−0.11*	0.22*	−0.03	−0.01	0.09*	0.11*
Substance-use index (ever use)	−0.09*	1.00	0.29*	−0.12*	0.15*	−0.17*	0.16*	−0.10*	0.27*	0.19*	−0.11*	−0.11*
Problem behaviors	−0.04*	0.29*	1.00	−0.10*	0.16*	−0.16*	0.17*	−0.05*	0.34*	0.29*	−0.07*	−0.09*
Positive well-being	0.35*	−0.12*	−0.10*	1.00	−0.48*	0.45*	−0.29*	0.39*	−0.11*	−0.17*	0.16*	0.18*
Hopelessness	−0.21*	0.15*	0.16*	−0.48*	1.00	−0.47*	0.42*	−0.27*	0.18*	0.24*	−0.10*	−0.12*
Self-esteem	0.17*	−0.17*	−0.16*	0.45*	−0.47*	1.00	−0.36*	0.32*	−0.18*	−0.25*	0.11*	0.12*
Anxiety / worry	−0.11*	0.16*	0.17*	−0.29*	0.42*	−0.36*	1.00	−0.18*	0.15*	0.23*	−0.08*	−0.08*
Social connectedness	0.22*	−0.10*	−0.05*	0.39*	−0.27*	0.32*	−0.18*	1.00	−0.07*	−0.14*	0.13*	0.14*
Sensation seeking	−0.03	0.27*	0.34*	−0.11*	0.18*	−0.18*	0.15*	−0.07*	1.00	0.31*	−0.09*	−0.08*
Boredom	−0.01	0.19*	0.29*	−0.17*	0.24*	−0.25*	0.23*	−0.14*	0.31*	1.00	−0.03	−0.04*
Friends’ disapproval of drugs	0.09*	−0.11*	−0.07*	0.16*	−0.10*	0.11*	−0.08*	0.13*	−0.09*	−0.03	1.00	0.53*
Parents’ disapproval of drugs	0.11*	−0.11*	−0.09*	0.18*	−0.12*	0.12*	−0.08*	0.14*	−0.08*	−0.04*	0.53*	1.00
Non-MSA												
Health behaviors	1.00	−0.13*	−0.06*	0.34*	−0.25*	0.18*	−0.14*	0.23*	−0.05*	−0.02	0.09*	0.10*
Substance-use index	−0.13*	1.00	0.31*	−0.16*	0.19*	−0.18*	0.18*	−0.11*	0.30*	0.23*	−0.13*	−0.15*
Problem behaviors	−0.06*	0.31*	1.00	−0.13*	0.20*	−0.18*	0.20*	−0.06*	0.38*	0.31*	−0.10*	−0.12*
Positive well-being	0.34*	−0.16*	−0.13*	1.00	−0.48*	0.41*	−0.30*	0.41*	−0.13*	−0.18*	0.16*	0.17*
Hopelessness	−0.25*	0.19*	0.20*	−0.48*	1.00	−0.46*	0.41*	−0.28*	0.21*	0.26*	−0.12*	−0.13*
Self-esteem	0.18*	−0.18*	−0.18*	0.41*	−0.46*	1.00	−0.35*	0.33*	−0.20*	−0.26*	0.12*	0.13*
Anxiety / worry	−0.14*	0.18*	0.20*	−0.30*	0.41*	−0.35*	1.00	−0.18*	0.18*	0.25*	−0.09*	−0.09*
Social connectedness	0.23*	−0.11*	−0.06*	0.41*	−0.28*	0.33*	−0.18*	1.00	−0.07*	−0.15*	0.13*	0.14*
Sensation seeking	−0.05*	0.30*	0.38*	−0.13*	0.21*	−0.20*	0.18*	−0.07*	1.00	0.35*	−0.10*	−0.09*
Boredom	−0.02	0.23*	0.31*	−0.18*	0.26*	−0.26*	0.25*	−0.15*	0.35*	1.00	−0.03	−0.04
Friends’ disapproval	0.09*	−0.13*	−0.10*	0.16*	−0.12*	0.12*	−0.09*	0.13*	−0.10*	−0.03	1.00	0.53*
Parents’ disapproval	0.10*	−0.15*	−0.12*	0.17*	−0.13*	0.13*	−0.09*	0.14*	−0.09*	−0.04	0.53*	1.00

Values are Spearman ρ;

* indicates p < 0.001.

**Table 4.** System-Level Network Metrics by MSA Status

Network metric	MSA	Non-MSA
Mean absolute correlation	0.202	0.213
Mean degree (number of connections)	7.33	7.50
Mean betweenness centrality	0.033	0.032
Mean eigenvector centrality	0.280	0.279

Threshold for edge inclusion: |ρ| > 0.10

**Table 5.** Node-Level Centrality Metrics by MSA Status

	MSA			Non-MSA		

Variable	Degree	Betweenness	Eigenvector	Degree	Betweenness	Eigenvector

Health behaviors	6	0.012	0.236	7	0.014	0.270
Substance-use index (ever use)	9	0.075	0.321	11	0.123	0.368
Problem behaviors	9	0.041	0.339	7	0.003	0.283
Positive well-being	11	0.140	0.379	11	0.123	0.368
Hopelessness	9	0.036	0.342	9	0.025	0.337
Self-esteem	9	0.040	0.337	9	0.044	0.325
Anxiety / worry	8	0.017	0.316	8	0.009	0.312
Social connectedness	3	0.000	0.119	3	0.000	0.118
Sensation seeking	6	0.003	0.251	7	0.011	0.271
Boredom	7	0.011	0.280	8	0.009	0.312
Friends’ disapproval of drugs	6	0.018	0.234	6	0.021	0.220
Parents’ disapproval of drugs	5	0.007	0.205	4	0.000	0.164

|ρ| > 0.10 adjacency; degree = number of connections
